# Pretreatment rate of decay in forced vital capacity predicts long-term response to pirfenidone in patients with idiopathic pulmonary fibrosis

**DOI:** 10.1038/s41598-018-24303-4

**Published:** 2018-04-13

**Authors:** Davide Biondini, Elisabetta Balestro, Donato Lacedonia, Stefania Cerri, Rosanna Milaneschi, Fabrizio Luppi, Elisabetta Cocconcelli, Erica Bazzan, Enrico Clini, Maria Pia Foschino Barbaro, Dario Gregori, Manuel G Cosio, Marina Saetta, Paolo Spagnolo

**Affiliations:** 1 Department of Cardiac, Thoracic and Vascular Sciences, University of Padova, Padova, Italy; 2 Department of Medical and Surgical Sciences, Institute of Respiratory Diseases, University of Foggia, Foggia, Italy; 3 Center for Rare Lung Diseases, University Hospital Policlinico of Modena, Modena, Italy; 4 Respiratory Disease Unit, Santa Maria della Misericordia University Hospital, Udine, Italy; 5 Respiratory Disease Unit, University Hospital of Modena, Modena, Italy; 6 Meakins Christie Lab, Respiratory Division, McGill University, Montreal, Canada

## Abstract

Pirfenidone reduces functional decline in patients with Idiopathic Pulmonary Fibrosis (IPF). However, response to treatment is highly heterogeneous. We sought to evaluate whether response to pirfenidone is influenced by the pretreatment rate of forced vital capacity (FVC) decline. Fifty-six IPF patients were categorized as rapid (RP) or slow progressors (SP) based on whether their FVC decline in the year preceding pirfenidone treatment was > or ≤ 10% predicted. Following pirfenidone treatment patients were followed-up every 6 months and up to 24 months. In the entire population, pirfenidone reduced significantly FVC decline from 231 to 49 ml/year at 6 months (T6) (p = 0.003) and this effect was maintained at the 12-, 18- and 24-month time points (p value for trend n.s.). In RP, the reduction of FVC decline was evident at 6 months (36 vs 706 ml/year pretreatment; p = 0.002) and maintained, though to a lesser degree, at 12 (106 ml/year), 18 (176 ml/year) and 24 months (162 ml/year; p value for trend n.s). Among SP, the reduction in FVC decline was not significant at any of the time points analyzed. In conclusion, pirfenidone reduces FVC decline in IPF patients. However, its beneficial effect is more pronounced in patients with rapidly progressive disease.

## Introduction

Idiopathic pulmonary fibrosis (IPF), the most common and severe of the idiopathic interstitial pneumonias (IIPs), is a chronic and relentlessly progressive disease of unknown origin with a median survival of 3–5 years from diagnosis^1^. While the overall prognosis of IPF is poor, its rate of decline and progression to death is highly variable with some patients remaining relatively stable over a prolonged period of time or progressing slowly, and others experiencing a rapid decline^2,3^. The heterogeneous nature of the disease makes it difficult, if at all possible, to foresee the clinical trajectory in individual patients. Several risk prediction models have been developed in IPF, but their predictive capability is only moderate^4–6^.

Forced vital capacity (FVC) is a reliable, valid and reproducible measure of disease progression in patients with IPF^1,7^, and change in FVC percentage predicted (FVC% pred.) over time is a well-established predictor of mortality. Indeed, it has been shown that patients who experience a decrease in FVC% pred. greater than 10% over a 12-month period have a significantly lower 5-year survival compared to patients whose FVC% pred. declines of 10% or less during the same period of time^8^. While there is no universally agreed upon definition for these two clinical phenotypes, they are commonly referred to as “rapid” and “slow” progressors, respectively. Notably, the observation that rapidly progressive and relatively stable/slowly progressive patients with IPF display distinct gene expression^9,10^ and inflammatory profiles in the lung parenchyma^3^ supports the notion that the mechanisms underlying these distinct clinical phenotypes may also be different.

Pirfenidone, a pyridone derivative with anti-inflammatory, anti-fibrotic and anti-oxidant properties, is approved worldwide for the treatment of IPF based on its ability to slow down functional decline and disease progression as shown in three phase III clinical trials^11–13^. Clinical trials however are usually performed in highly selected patient populations and in clinical settings that reflect only partially real-life clinical practice. Patients enrolled in clinical trials in fact tend to be younger, and have less severe disease and fewer comorbidities, with a shorter follow-up. Yet, reassuringly, outcomes of IPF patients treated with antifibrotic drugs (e.g., pirfenidone and nintedanib) in real-life appear to be comparable to those observed in clinical trials^14^.

In this longitudinal study, we aimed to assess long-term (24 months) response to pirfenidone treatment in a well-characterized cohort of patients with IPF in a real-life setting. The availability of data from patients followed up before the pirfenidone era gave us the unique opportunity to stratify our patient population in rapid and slow progressors based on their rate of FVC decline in the 12-month pretreatment period.

## Results

A total of 56 patients were included in the study. Baseline patient demographics and clinical characteristics are summarized in Table 1. Most patients were males (78%) and ex-smokers (71%), with a median age at diagnosis of 67 years (range 37–78). Based on the annual FVC% pred. decline in the pretreatment period, 39 patients were classified as slow (FVC% pred. ≤10%) and 17 as rapid progressors (FVC% pred. >10%). Gender, age at diagnosis, smoking history and functional impairment (as assessed by FVC and DL_CO_) were similar in the two groups. Notably, a trend towards a higher FVC% pred. at diagnosis was seen in rapid progressors, consistent with previous publications from our group^3^. Pulmonary function data were available for all patients at the 12-month follow-up, and for most of them at 24 months (38 out of 56, 68%). Eighteen patients did not complete the study due to missed follow-up visits (n = 7), pirfenidone-related adverse effects (n = 2), lung transplantation (n = 3) or death (n = 6). Causes of death were related to chronic respiratory deterioration caused by IPF (n = 5) and left heart failure (n = 1). No acute exacerbations occurred in the entire study population; one possible explanation could be related to the selection bias as patients had to have a long (i.e. at least one year) follow-up without acute exacerbations before initiating pirfenidone treatment.

**Table 1:** Demographics and clinical characteristics of the entire population (n = 56), slow progressors (n = 39) and rapid progressors (n = 17).

	Entire population (n=56)	Slow progressors (n=39)	Rapid progressors (n=17)	p value
Male - *n (%*)	44 (78%)	31 (79%)	13 (76%)	0.8
Age at diagnosis - *years*	67 (37–78)	67 (37–78)	67 (54–77)	0.8
Former smokers - *n (%*)	40 (71%)	28 (72%)	12 (71%)	0.9
Smoking history - *Pack-Years*	10 (0–60)	8.5 (0–60)	15 (0–60)	0.4
Radiologic diagnosis - *n (%*)	37 (66%)	24 (62%)	13 (76%)	0.3
FVC at diagnosis - *L*	2.66 (1.19–4.72)	2.72 (1.19–4.72)	2.66 (1.69–3.79)	0.5
FVC at diagnosis - *%pred*.	80 (35–116)	74 (35–116)	83 (61–105)	0.1
DL_CO_ at diagnosis - *%pred*.	54 (28–114)	52 (28–114)	61 (48–75)	0.1
Transplanted patients - *n*	3	3	0	0.5
Deaths - *n*	6	5	1	0.4

Values are expressed as numbers and (%) or median and (ranges).

**Figure 1:**
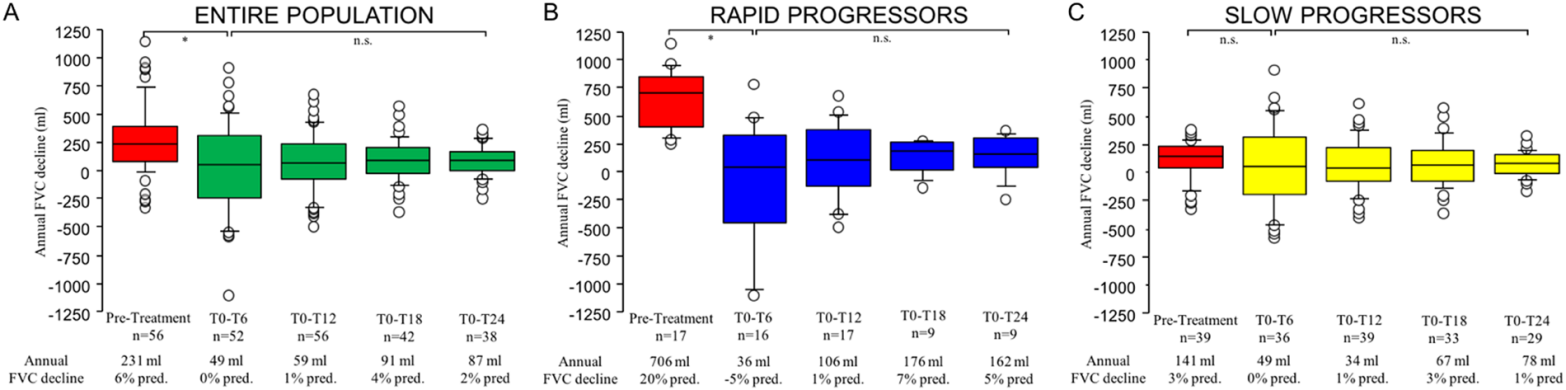
Panel (A) Annual FVC decline in the entire population (n = 56) before and after pirfenidone treatment at 6 (T0-T6), 12 (T0-T12), 18 (T0-T18) and 24 (T0-T24) months. Overall comparison between all time points was performed using the repeated measurements analysis of variance (ANOVA) (p = 0.03). Pirfenidone reduced significantly the annual decline in FVC already at 6 months (paired t-test, p = 0.003) and this reduction was maintained at 12-, 18- and 24-month follow-up (repeated measures analysis of variance at all time points, p = n.s.). Panel (B) Annual FVC decline in the rapid progressors (n = 17) before and after pirfenidone treatment at 6 (T0-T6), 12 (T0-T12), 18 (T0-T18) and 24 (T0-T24) months. Overall comparison between all time points was performed using the repeated measurements analysis of variance (ANOVA) (p < 0.001). Pirfenidone reduced significantly the annual decline in FVC already at 6 months (paired t-test, p < 0.01) and this reduction was maintained at 12-, 18- and 24-month follow-up (repeated measures analysis of variance at all time points, p = n.s.). Panel (C) Annual FVC decline in the slow progressors (n = 39) before and after pirfenidone treatment at 6 (T0-T6), 12 (T0-T12), 18 (T0-T18) and 24 (T0-T24) months. Overall comparison between all time points was performed using the repeated measurements analysis of variance (ANOVA) (p = 0.1). Pirfenidone did not significantly reduce the annual decline in FVC at any of the time points examined (paired t-test, p = n.s.; repeated measures analysis of variance at all time points, p = n.s.). Negative values mean improvement of FVC. Horizontal bars represent median values, bottom and top of each box plot represents 25^th^ and 75^th^ percentiles, brackets 10^th^ and 90^th^ percentiles, while circles represent outliers. *p value < 0.01, n.s. non significant.

In the pretreatment period, three patients received low-dose steroids, and one N-acetyl cysteine. In the pretreatment period, the median rate of annual FVC decline was 231 ml (range −323 to 1140 ml) in the entire population, 141 ml (range −323 to 375 ml) in slow progressors and 706 ml (range 242 to 1141 ml) in rapid progressors (Fig. 1A–C).

### Disease progression in the entire IPF population during treatment

In the IPF population as a whole (n = 56), pirfenidone treatment reduced significantly the rate of annual FVC decline at all the time points examined compared to the pretreatment period. Specifically, the median rate of annual FVC decline changed from 231 ml/year (corresponding to 6% pred./year) in the pretreatment period to 49 ml/year at 6 months (T6) (0% pred., p = 0.003), and this reduction persisted at 12- (59 ml/year, 1% pred.), 18- (91 ml/year, 4% pred.) and 24-month follow-up (87 ml/year, 2% pred.) (p value for trend from T6 to T24 n.s.) (Fig. 1A).

Similar results were observed when the patient populations from Padua, Modena, Foggia and Udine were analyzed separately (data not shown).

### Effect of pirfenidone treatment in rapid and slow progressors

**Figure 2:**
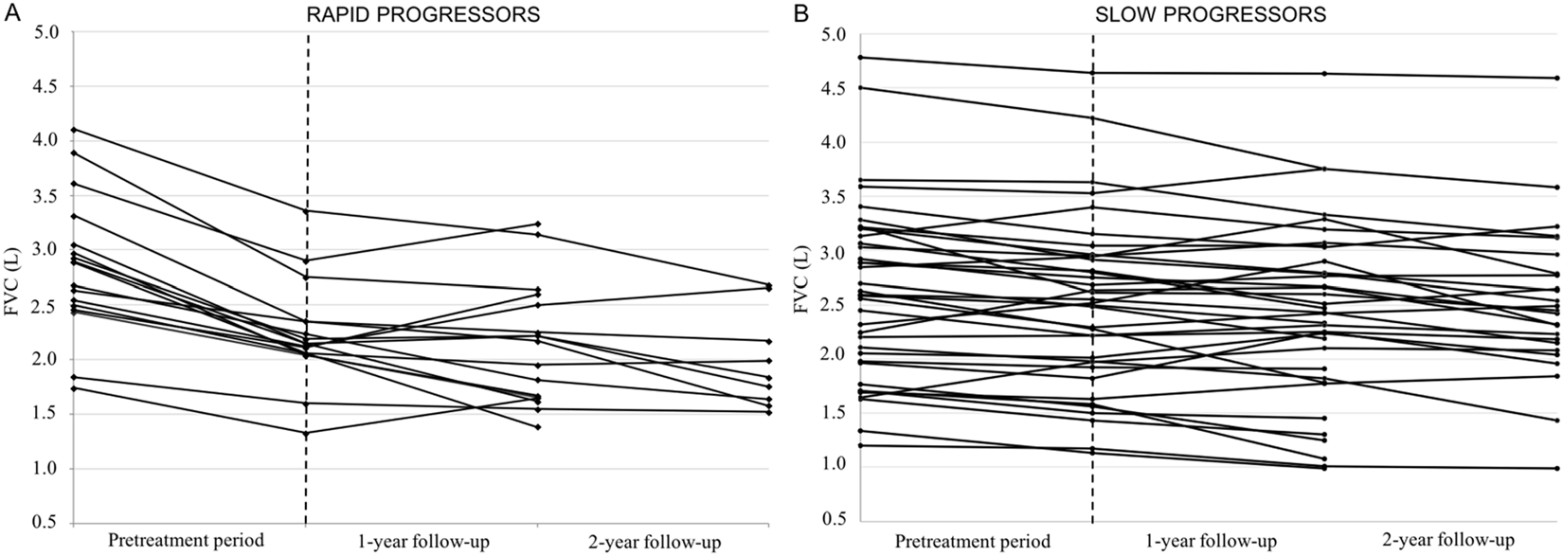
Panel (**A**) Individual FVC trajectories in rapid progressors from pretreatment period through 2-year follow-up. Overall comparison between all time points was performed using the repeated measurements analysis of variance (ANOVA) (p < 0.001). In the pretreatment period, the FVC trajectory of the rapid progressors is depicted by a line with a steep slope. Whereas, after the institution of pirfenidone (vertical dotted line), FVC trajectory is depicted by a line with a significantly flatter slope compared to the pretreatment period (paired t-test, p < 0.01). The FVC values during treatment were stable in the 2-year follow-up (repeated measures analysis of variance at 1-year and 2-year time points, p = n.s.). (**B**) Individual FVC trajectories in slow progressors from pretreatment period through 2-year follow-up. Overall comparison between all time points was performed using the repeated measurements analysis of variance (ANOVA) (p = n.s.). In the pretreatment period, the FVC trajectory of the slow progressors is depicted by a line with a flat slope. After the institution of pirfenidone (vertical dotted line), FVC trajectory is depicted by a line with a slope as flat as the pretreatment period (paired t-test, n.s.). The FVC values during treatment were stable in the 2-year follow-up (repeated measures analysis of variance at 1-year and 2-year time points, p = n.s.). FVC (on the y axis) is expressed as litres. *p value < 0.01, n.s. non significant.

When rapid progressors (n = 17) and slow progressors (n = 39) were analyzed separately, the magnitude of pirfenidone effect on FVC decline varied greatly (Fig. 1B,C and Fig. 2A,B). Indeed, among rapid progressors the beneficial effect of pirfenidone on FVC decline was already evident at 6 months (T6), wherein the annual rate of FVC decline changed from 706 ml/year (20% pred.) in the pretreatment period to 36 ml/year (−5% pred., p = 0.002). The treatment effect was maintained at the 12- (106 ml/year, 1% pred.), 18- (176 ml/year, 7% pred.) and 24-month follow-up (162 ml/year, 5% pred.) (p value for trend from T6 to T24 n.s.) (Fig. 1B).

Among slow progressors, the reduction of the annual FVC decline compared to the pretreatment period did not reach statistical significance at any of the time points examined, i.e. at 6 months (T6) (49 ml/year, 0% pred. vs. 141 ml/year, 3% pred. pretreatment, p = 0.3) nor in any of the other follow-up time points (34 ml/year, 1% pred. at 12 months; 67 ml/year, 3% pred. at 18 months; 78 ml/year, 1% pred. at 24 months) (p value for trend from T6 to T24 n.s.) (Fig. 1C).

## Discussion

Pirfenidone is approved worldwide for the treatment of IPF based on its ability to slow down functional decline and disease progression^11–13^. The heterogeneous nature of IPF makes it difficult to foresee the clinical trajectory in individual patients, and this heterogeneity could conceivably also affect treatment response. In the present study, we report on the long-term (up to 24 months) efficacy of pirfenidone treatment in patients with IPF. These patients had been followed for a prolonged time before starting anti-fibrotic treatment, during which we had the opportunity to categorize them as rapid or slow progressors based on the rate of their FVC decline in the pretreatment period^3^. In our patient population as a whole, compared to the pretreatment period, pirfenidone reduced significantly the decline in FVC already at 6 months, and this reduction was maintained at the 12-, 18- and 24-month follow-up. However, the effect of pirfenidone differed considerably between slow and rapid progressors, being significantly more pronounced in the latter group at all time points.

Before starting pirfenidone, our patients were followed-up for a median of 15 months, and this gave us the possibility to monitoring changes in FVC during an extended period of time, which, in turn, provided a reliable basis for the definition of rapid and slow disease progression. FVC is a valid and reproducible measure of disease progression in patients with IPF^1,7,15^. IPF patients who experience a decrease of >10% in FVC% pred. over a 12-month period (rapid progressors) display a significantly lower 5-year survival than patients whose FVC% pred. declines ≤10% (slow progressors)^8^. Given the differences in the described rates of decay in IPF, it is conceivable that treatment response might also differ in subjects with rapid and slow decline. Accordingly, it is important to prospectively investigate this possibility.

When we considered our whole population together, the annual rate of FVC decline in the pretreatment period was 231 ml/year (6% pred.) similar to what has been reported in the placebo arms of other treatment trials^13^. With pirfenidone, the rate of FVC decline was variably but significantly reduced already after 6 months of treatment, an improvement that was maintained during the entire 24-month study duration. Overall, after one year of pirfenidone treatment, 7 (12%) patients experienced a FVC decline >10% compared with 17 patients (30%) in the pretreatment period (OR 3.0, p = 0.02), corresponding to a relative reduction of 59%, while the absolute fall of FVC decreased from a pretreatment value of 231 ml/year to 59 ml/year (p < 0.05), consistent with a previous study in Japanese patients^16^. Our data provide, we believe, a reliable picture of the effect of pirfenidone treatment on the rate of FVC change between the pretreatment and follow-up period, since each patient served as its own control, which clearly strengthens our findings.

The effect of pirfenidone differed significantly between slow and rapid progressors. Among rapid progressors, the median decline of FVC prior to treatment was 706 ml/year, which was reduced to 36 ml/year after 6 months of treatment. Overall, the pirfenidone effect was significantly maintained throughout the two-year study period. Pirfenidone was also beneficial for patients with slow pretreatment decline since, although the rate of FVC decline did not change significantly, it seemed to stabilise the disease.

Our results are in line with those of previous retrospective analyses of lung function changes in patients with mild to moderate IPF treated with pirfenidone^17,18^. These studies showed that patients with progressive disease (e.g., FVC% pred. decline > 10% per year) may benefit substantially from pirfenidone treatment, which may even result in improvement of FVC, as shown by Loeh *et al*.^18^, while patients with slowly progressive disease tend to experience disease stability under treatment. The assessment of disease progression and treatment response in patients with IPF is complicated by its variable clinical course^2^. In a recent post-hoc analysis of patients from the placebo arms of the CAPACITY and ASCEND trials^19^, Nathan and colleagues observed a weak negative correlation between changes in FVC% pred. during two consecutives 6-month intervals, that the authors interpreted as a reflection of the variability in both the magnitude and direction of change. An important source of variability in that study could have been the combined analysis of slow and rapid progressors together, particularly since the follow-up period was relatively short (i.e., 6 months). In our study, the median pretreatment observation period was 15 months, which minimizes the possibility that the observed rate of disease progression was confounded by the inherent intra-individual variability in longitudinal change in FVC.

A recent multicentre study^20^ confirmed the efficacy of pirfenidone in slowing down disease progression in patients with IPF, but suggested that the beneficial effect would be more pronounced in patients with more severe disease. This was not the case in our study, since at diagnosis the severity of the disease in our population was similar in rapid and slow progressors.

The mechanisms through which rapid progressors display a particularly favorable response to pirfenidone treatment is not known but may be related to differences in the described lung pathology in the slow and rapid IPF groups^3^. Possibly, extracellular matrix deposition and removal may be much more rapid, thus amenable to anti-fibrotic therapy, in the rapid progressive than in relatively stable IPF^18,21^. Recently, we have shown that explanted lungs from IPF subjects had similar degree of fibrosis deposition and numbers of fibroblast foci in slow and rapid progressors^3^. This finding might be due to a more rapid fibrous tissue deposition in the rapid decliner perhaps more amenable to lysis and degradation by pirfenidone. Alternatively, pirfenidone might play a prominent immunosuppressive and antioxidant role, with the antioxidant properties contributing to its anti-inflammatory effects, which in turn may account, at least in part, for pirfenidone’s antifibrotic effects^22,23^. We have reported that rapidly progressive IPF, compared to slow or stable disease, is characterized by a significantly higher innate and adaptive inflammation in the lung parenchyma. This might contribute significantly to the accelerated decline of this group of patients and would also make the disease more responsive to a compound with anti-inflammatory and anti-fibrotic properties^3,24^.

The presence of an exuberant immune inflammatory infiltrate, found predominantly in the rapid progressors, is consistent with the gene expression profile reported by Boon and colleagues^10^, who indeed described the activation of important pro-inflammatory pathways that may potentially play a role in the immune activation and disease progression in rapid decliners. In line with these reports it is also the presence of B cell aggregates^25^ and highly differentiated circulating B cells in patients with IPF^26^, findings usually observed in autoimmune syndromes. Our findings of an increased number of B lymphocytes and T lymphocytes in the lungs of rapid progressors^3^ are also in keeping with previous observations showing that lymphocyte density in IPF lung is associated with FVC decline and poor survival^27^. Although speculative and based on pathological description of the disease, we believe these possibilities deserve some attention since they might explain, at least in part, the different response to pirfenidone treatment.

The rate of discontinuation of pirfenidone due to adverse events observed in our patient population (3.5%) was lower than that observed in the CAPACITY^12^ (14.8%) and ASCEND^13^ (14.4%) trials, confirming the safety and tolerability of the drug in clinical practice.

Our long-term prospective study, even if relatively small, provides further evidence of the efficacy of pirfenidone in patients with IPF up to 24 months of treatment and shows that response to therapy is influenced by the rate of decline, slow or rapid, in the pretreatment period. Strengths of our study include the careful patient characterization and the availability of long-term pre- and post-treatment data. Owing to the progressive nature of the disease and the availability worldwide of two efficacious anti-fibrotic drugs (pirfenidone and nintedanib), studies on IPF patients off-treatment will become progressively less common, if at all possible (and ethical). This makes our data regarding the pretreatment period particularly relevant.

Our study has some limitations. Firstly, the size of our study population is relatively small, and it decreased over time; indeed, follow-up data at 24 months were available for 38/56 patients only (68%). Secondly, we limited our analysis to change in FVC. Despite uncertainties about its clinical meaningfulness and handling of missing data, FVC is widely accepted as surrogate of treatment efficacy in IPF both in clinical trials and at regulatory level^8,15,28,29^. Contrary to clinical trials however, we decided neither to impute missing values for patients who stopped pirfenidone treatment at some point during the observation period nor to set FVC as 0 for patients who died or underwent lung transplantation, as previously done^13^.

## Conclusions

The study confirms that pirfenidone treatment reduces significantly the rate of FVC decline in patients with IPF, an effect that is significantly more pronounced in patients with rapidly progressive disease. Additional studies are required to identify more precisely the underlying differences in disease behavior and treatment response of the slow and rapid decliners in IPF. This will greatly benefit clinical research and daily clinical practice alike.

## Methods

### Patients and study design

Informed consent was obtained for all study participants. This was a prospective, longitudinal, multicenter study, in which we analyzed a unique and well-characterized cohort of patients with IPF, with a long clinical and functional follow-up before and after the initiation of pirfenidone treatment. Fifty-six patients were selected from four Italian Interstitial Lung Disease (ILD) centers (e.g., University Hospital of Padua, n = 27; University Hospital of Foggia, n = 14; University Hospital of Modena, n = 10; and General Hospital of Udine, n = 5). For all patients, the diagnosis of IPF was made in accordance with current guidelines^1^.

The peculiarity of this study was to include only patients for whom lung function data were available for at least one year before (*pretreatment period*) starting pirfenidone treatment. Based on their annual rate of decline in FVC% pred. in the pretreatment period, patients were classified as either “rapid” (decline in FVC% pred. >10%) or “slow” (decline in FVC% pred. ≤10%) progressors. They were then followed-up during pirfenidone treatment every 6 months up to 24 months (*follow-up period*). At the 12-month follow-up, functional data were available for the entire patient population, whereas at 24 months functional data were available for 38/56 (68%) patients.

Negative values of annual FVC decline during the follow-up indicated amelioration. The study was performed in accordance with the Declaration of Helsinki and was approved by the Ethics Committee of the University Hospital of Padua (4280/AO/17).

Additional important clinical and functional parameters, including symptoms, diffusing capacity of the lung for carbon monoxide (DL_CO_) and 6-minute walking test, were also collected but the amount of missing data did not allow for a meaningful statistical analysis to be performed.

### Statistical analysis

Categorical variables were described as absolute (n) and relative values (%) and continuous variable were described as median and range. To compare demographic data and baseline clinical characteristics between rapid and slow progressors Chi-square test for categorical variables and Mann-Whitney test for the continuous variables were used.

In the entire population, as well as in the rapid and slow progressor subgroups, we performed the repeated measurements analysis of variance (ANOVA) at all time points to evaluate the difference in FVC decline between pretreatment and the follow-up period. To evaluate the difference between the pretreatment FVC decline and the first time point available (6 months) in the follow-up period in the entire population, in the rapid progressors and in the slow progressors we performed a paired t-test analysis. Finally, in order to evaluate whether this potential difference was maintained in the follow-up period (6, 12, 18 and 24 months), we performed the repeated measurements analysis of variance (ANOVA) between these time points in the entire population, and in the rapid and slow progressor subgroups.

All data were analysed using SPSS Software version 22.0 (IBM USA). p-values < 0.05 were considered statistically significant.

### Data availability

All data generated or analyzed during this study are included in this published article.
